# The Feasibility, Safety, and Preliminary Functional Outcomes of a Mobile Application-Based Rehabilitation Program in Non-Ambulatory Patients After Intensive Care Unit Discharge

**DOI:** 10.3390/jcm15114211

**Published:** 2026-05-29

**Authors:** Seungwoo Cha, Ye Ji Kim, Chaelin Lee, Yong Hoe Koo, Sanghee Lee, Jaeho Choi, Young-In Yoon, Kyung-Wook Jo, Youngran Lee, Won Kim

**Affiliations:** 1 Department of Rehabilitation Medicine, Asan Medical Center, University of Ulsan College of Medicine, Seoul 05505, Republic of Korea; 2 Asan Institute for Life Sciences, Asan Medical Center, University of Ulsan College of Medicine, Seoul 05505, Republic of Korea; 3 EverEx Inc., Seoul 06628, Republic of Korea; 4 Division of Hepatobiliary Surgery and Liver Transplantation, Department of Surgery, Asan Medical Center, University of Ulsan College of Medicine, Seoul 05505, Republic of Korea; 5 Division of Pulmonology and Critical Care Medicine, Department of Internal Medicine, Asan Medical Center, University of Ulsan College of Medicine, Seoul 05505, Republic of Korea; 6 Department of Nursing, Asan Medical Center, Seoul 05505, Republic of Korea

**Keywords:** intensive care unit-acquired weakness, mobile health, digital rehabilitation, early mobilization, exercise

## Abstract

**Background:** Although early mobilization has been shown to improve clinical outcomes after intensive care unit (ICU)-acquired weakness, its implementation remains limited in routine clinical practice. This study aimed to evaluate the feasibility, safety, and preliminary clinical outcomes of a mobile application-based rehabilitation program in non-ambulatory patients during the early ward phase following ICU discharge. **Methods:** This prospective single-arm pilot trial included adult patients (≥19 years) who had received ICU care and demonstrated limited ambulatory function, defined as Functional Ambulatory Category (FAC) ≤3. Participants received an individualized, application-guided exercise program comprising two daily sessions over two weeks. Primary outcomes were programmatic feasibility, safety, and patient satisfaction. Rehabilitation compliance was quantified using application usage logs and categorized as high (≥50%) or low (<50%). Secondary functional outcomes, such as Medical Research Council Sum Score (MRC-SS), ICU Mobility Scale, FAC, muscle strength measures, health-related quality of life, and pain scores, were assessed at baseline, week 1, and week 2. **Results:** Of the 25 initially enrolled patients, 5 dropped out due to clinical status changes or transfers, yielding a retention rate of 80.0%. For the 20 analyzed patients (mean age 52.7 ± 13.9 years; 45% male), the overall mean rehabilitation compliance was 40.6%. No serious adverse events related to the intervention were reported, and overall patient satisfaction and application usability were high. Progressive increases in exercise intensity and training levels were observed throughout the intervention period. Significant improvements over time were found in MRC-SS, ICU Mobility Scale, FAC, grip strength, health-related quality of life, and pain scores (all *p* < 0.05). Although compliance-based recovery trajectories were confounded by small subgroup sizes and baseline clinical imbalance, exploratory analyses nonetheless identified statistically significant time × compliance interaction effects for MRC-SS and straight leg raise performance. **Conclusions:** This pilot study demonstrates that a mobile application-based rehabilitation program is a feasible and safe approach to implement in deconditioned patients after ICU discharge. These preliminary functional recovery trajectories provide encouraging signals, suggesting that this digital platform may serve as a potential adjunct to conventional care. Rigorous, randomized controlled trials are required to confirm its definitive clinical efficacy and scalability.

## 1. Introduction

Intensive care unit-acquired weakness (ICUAW) is a common complication among critically ill patients, particularly those who experience prolonged mechanical ventilation, sepsis, or multi-organ dysfunction [1]. ICUAW encompasses critical illness myopathy and polyneuropathy and is associated with increased mortality, prolonged use of mechanical ventilation, extended intensive care unit (ICU) and hospital stay, and persistent functional impairment [2,3]. Notably, the consequences of ICUAW are not confined to the acute phase of critical illness; affected patients often experience long-term functional impairment, with significantly reduced physical function and quality of life persisting up to five years after ICU discharge [4]. Established risk factors include prolonged immobility, hyperglycemia, and exposure to corticosteroids or neuromuscular blocking agents [3].

Early mobilization and therapeutic exercise have been shown to reduce the incidence of ICUAW, enhance functional independence, and shorten the duration of mechanical ventilation and delirium [5,6,7]. However, recovery from ICUAW requires sustained rehabilitation that extends beyond the ICU stay into the post-ICU ward phase. This ongoing deficit is closely tied to post-intensive care syndrome, which encompasses long-term physical, cognitive, and mental health impairments that persistently compromise survivors’ quality of life [8]. Despite this, many patients with ICUAW remain largely bedridden after transfer to general wards [9], as they are often encumbered by medical devices, catheters, and monitoring equipment. Additionally, concerns regarding fall risk frequently limit independent ambulation, resulting in prolonged periods of inactivity in bed [10]. Consequently, although ongoing rehabilitation is clearly warranted, many healthcare systems struggle to deliver rehabilitation with sufficient intensity and frequency during this transitional period. These gaps in care underscore the need for personalized, bedside rehabilitation programs that patients can perform safely and independently. While various post-ICU clinic models have been proposed to manage the multifaceted challenges of recovery, their widespread implementation often faces significant resource constraints and organizational barriers [11].

In this context, digital health technologies have emerged as promising adjuncts to conventional rehabilitation delivery. Digital therapeutics, particularly mobile application-based rehabilitation, offer scalable, low-resource tools for guiding therapeutic exercise, monitoring performance, and promoting patient engagement without the need for continuous direct therapist supervision [12,13]. Recent studies have demonstrated that mobile applications delivering personalized exercise videos can improve physical function and increase confidence in exercise performance among patients requiring physical rehabilitation [14]. Moreover, in the post-ICU phase, where access to formal rehabilitation services is often reduced, mobile health interventions may help bridge this gap by ensuring continuity of care. These platforms enable the delivery of tailored, structured exercise protocols that patients can perform at the bedside, thereby addressing both physical barriers (e.g., immobility) and organizational constraints (e.g., staffing shortages). Crucially, rather than replacing hands-on physiotherapy, these digital solutions serve as a practical adjunct within a hybrid model—maximizing rehabilitation volume and optimizing limited clinical resources by enabling safe, independent training between formal sessions. Despite these advantages, robust clinical evidence regarding the efficacy of mobile application-based rehabilitation specifically for patients with ICUAW during the transition from ICU to general wards remains limited, highlighting the need for more rigorous evaluation.

In this context, we developed a structured rehabilitation program delivered via a mobile application designed to guide ICU survivors through individualized mobility exercises. The objective of this study was to evaluate the feasibility, safety, and preliminary clinical outcomes of this mobile application-based rehabilitation program for improving functional recovery in critically ill patients during the early post-ICU ward phase.

## 2. Methods

### 2.1. Study Population

This study was conducted as a prospective, single-arm clinical trial at a single tertiary referral center. Adult inpatients (≥19 years) who had recently been discharged from the ICU and remained non-ambulatory during the early ward phase were screened for eligibility. Eligibility required a Functional Ambulatory Category (FAC) score of ≤3, indicating that patients required physical assistance or supervision and were unable to ambulate independently. Patients were excluded if they were unable to follow the mobile application-based rehabilitation program due to cognitive impairment; had severe pain or musculoskeletal limitations precluding participation; were medically unstable; or had significant visual or auditory impairments that would interfere with program adherence. Rather than utilizing objective cutoff scores for these impairments, exclusion was determined through a clinical assessment focused on the practical usability of the mobile application, ensuring that only those with deficits severe enough to preclude program participation were excluded.

A formal sample size calculation was not performed given the pilot nature of this study. Instead, a target sample size of 20 participants was determined based on practical feasibility and established conventions in the existing literature. This size is consistent with prior pilot trials evaluating post-ICU rehabilitation and mobile health interventions, which included a similar number of participants that is sufficient to assess preliminary safety, compliance, and protocol feasibility before conducting a definitive large-scale trial [15,16].

The study was conducted in accordance with the principles of the Declaration of Helsinki and was approved by the Institutional Review Board of Asan Medical Center (IRB No. 2024-1160). The trial was registered at ClinicalTrials.gov (NCT06849765). Before enrollment, written informed consent was obtained from all participants or their legally authorized representatives after a detailed explanation of the study objectives, procedures, potential risks, and benefits. Participation was voluntary, and participants were informed of their right to withdraw from the study at any time without any impact on their clinical care.

### 2.2. Intervention

Eligible patients received an individualized rehabilitation program delivered through the MORA digital rehabilitation system (version 1.2; EverEx Inc., Seoul, Republic of Korea), which comprised a clinician-facing web portal (MORA-Ex) and a patient-facing mobile application (MORA). Through the web-based portal, treating clinicians constructed personalized exercise programs using a rehabilitation exercise library comprising over 3000 exercises. Clinicians also monitored patient participation through platform-generated usage data and retained full authority to select, modify, or adjust exercises throughout the intervention. Prescribed programs were synchronized to the patient’s mobile application, allowing participants to perform assigned exercises using their smartphones. The application delivered structured exercise sessions via video-guided demonstrations accompanied by brief on-screen instructions to facilitate accurate movement execution. Before initiating the intervention, each participant underwent a standardized functional screening assessment to determine feasible movements across different postures (supine, active bed-level, and seated positions). Based on this assessment, rehabilitation physicians prescribed personalized exercise programs with difficulty levels tailored to each participant’s posture and functional capacity. The intervention protocol comprised stepwise exercise modules targeting the upper extremities, lower extremities, and trunk (Supplementary Materials). To ensure standardization of the exercise stimulus despite individual tailoring, each participant was required to perform a minimum set of core exercises within each module, categorized by functional steps. Step 0 included passive-to-assisted and low-intensity bed-level mobility exercises; Step 1 comprised active bed-level strengthening exercises; and Step 2 consisted of seated functional and strengthening exercises. Resistance was applied using sandbags when participants were able to perform movements with additional weight. Sandbags of 0.5 kg, 1.0 kg, and 1.5 kg were used for upper extremity exercises, and 0.5 kg, 1.0 kg, 1.5 kg, and 2.0 kg were used for lower extremity exercises to ensure individualized intensity.

**Figure 1:**
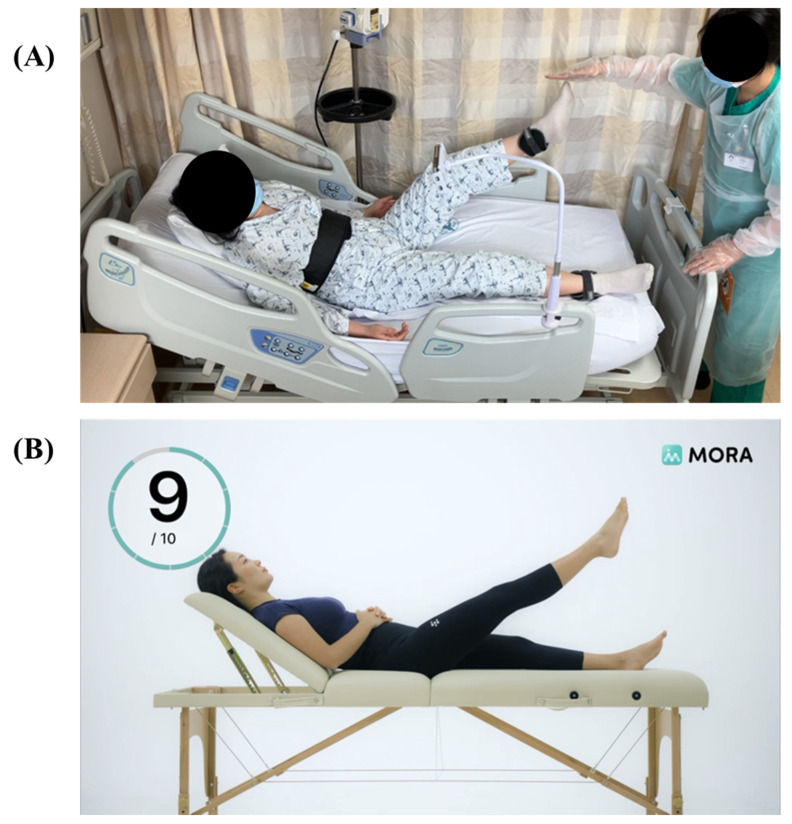
Application-guided inpatient rehabilitation using the MORA platform. (**A**) A hospitalized patient performing a lower extremity exercise under the direct supervision of a physical therapist while following exercise guidance provided by the MORA. (**B**) A screenshot of the MORA interface demonstrating standardized exercise instructions.

Treating clinicians were permitted to modify prescribed exercise components by adding or excluding specific movements based on clinical judgment, safety considerations, and individual patient conditions, including medical stability, fatigue level, pain, and cardiopulmonary tolerance. All exercise prescriptions were reviewed and adjusted as needed throughout the intervention period to maintain appropriateness and patient safety. During the first five days of the intervention, a trained physical therapist conducted in-person visits to confirm proper application use and correct execution of exercise movements (Figure 1). Thereafter, participants were encouraged to perform the program with caregiver supervision, supported by regular reminders delivered via application push notifications, telephone calls, and text messages. Progression between steps and the application of resistance (sandbags) was determined based on each participant’s ability to complete movements without significant fatigue or pain. Daily adherence to the prescribed exercise sessions was monitored through application logs and direct verification.

The intervention protocol comprised two daily sessions (morning and afternoon), with a planned total of 20 sessions over a two-week period. If participants were unable to complete the full protocol because of a clinical condition or temporary limitations, the intervention period was extended up to a maximum of four weeks to allow for completion. Each session comprised 10–15 exercise movements and was designed to be completed within approximately 20 min. Collectively, while the specific exercise selection was tailored from the digital library, the progression logic and core muscle group targets remained strictly standardized across all participants to ensure a consistent intervention structure.

During the study period, all participants received standard acute ward care and conventional rehabilitation services following ICU discharge. Based on established multi-disciplinary frameworks for post-ICU care, usual care comprised routine nursing mobilization—including vital sign monitoring, position changes, and assistance with basic bedside activities—alongside standard physical therapy consisting of general range-of-motion exercises and progressive ambulation training tailored to the post-transplantation status [17,18]. The mobile application-based program was implemented not as a replacement for standard care, but as a structured, low-resource adjunct designed to overcome institutional barriers by facilitating independent, high-frequency bedside exercises.

### 2.3. Clinical Characteristics and Outcomes

Baseline demographic and clinical characteristics were collected at enrollment and included age, sex, body mass index, vital signs, primary diagnosis, surgical or interventional history, and comorbidities. Indicators of illness severity and hospitalization course were also recorded, including length of hospital stay, ICU admission and duration, use and duration of mechanical ventilation, laboratory findings, and the presence of a caregiver during hospitalization.

The primary focus of this pilot trial was to evaluate the clinical feasibility and implementation safety of the mobile application-based rehabilitation program in the post-ICU general ward environment. Feasibility was quantified using three programmatic metrics: retention rate, compliance, and patient-reported usability and satisfaction. Retention rate was defined as the percentage of enrolled participants who successfully completed the final 2-week follow-up assessments. Compliance was quantified using automated usage logs generated by the MORA. It was calculated as the proportion of completed independent sessions relative to the total number of prescribed sessions during the 2-week intervention period, excluding initial sessions conducted jointly with a physical therapist. Participants were categorized into high-compliance (≥50%) and low-compliance (<50%) groups based on this metric, where the 50% threshold ensured a pragmatic minimum exposure of at least one session per day, which aligns with the reported mean adherence rate of approximately 56% in previous mHealth research [19]. Patient-reported usability and satisfaction were evaluated at Week 1 and Week 2 using a standardized 5-point Likert scale questionnaire (ranging from 1 = strongly disagree to 5 = strongly agree) covering overall satisfaction, perceived effectiveness, application-related experience, and safety. Perceived exercise intensity was also scored from 1 (very easy) to 5 (very difficult). Safety was continuously monitored throughout the study period and quantified by the incidence of intervention-related adverse events, such as falls, dizziness, hemodynamic instability, or device-related complications. In addition, physiological tolerance was monitored via heart rate reserve (%HRR), calculated as (peak heart rate during assessment − resting heart rate)/(220 − age − resting heart rate) × 100 [20], where resting heart rate was measured immediately before and peak heart rate during the functional assessment.

Functional status and physical performance were assessed at three time points: enrollment (baseline, Week 0), Week 1, and Week 2. Outcome measures included the Medical Research Council Sum Score (MRC-SS), ICU Mobility Scale, FAC, Sit-to-Stand test, Straight Leg Raise (SLR) test, bridge performance assessment, hand grip strength, knee extensor strength, and the 10 m walk test. The MRC-SS evaluates bilateral shoulder abduction, elbow flexion, wrist extension, hip flexion, knee extension, and ankle dorsiflexion on a 0–5 ordinal scale, yielding a total score ranging from 0 to 60, with higher scores indicating greater global muscle strength [21]. A difference of more than 4 points on the MRC-SS is established as the minimal clinically important difference [22]. Mobility level was assessed using the ICU Mobility Scale, an ordinal scale ranging from 0 (lying in bed) to 10 (independent ambulation), reflecting the highest level of mobility achieved during assessment [23]. An improvement of 1.0 to 3.0 points on this scale has been validated as a clinically meaningful change in post-ICU populations, depending on the methods applied [24]. The FAC categorizes walking ability according to the degree of physical assistance required, ranging from 0 (non-functional ambulation) to 5 (independent ambulation on all surfaces) [25]. Scores of 1 and 2 indicate the need for continuous or intermittent manual contact, respectively; a score of 3 indicates the need for supervision without physical assistance; and scores of 4 and 5 reflect increasing levels of independence. The SLR test was graded using a 4-level ordinal scale: grade 0, the inability to actively elevate the leg; grade 1, active elevation < 30°; grade 2, active elevation ≥ 30° with fewer than 10 repetitions; and grade 3, active elevation ≥ 30° with 10 or more repetitions. Bridge performance was graded similarly: grade 0, the inability to perform bridging; grade 1, elevation sufficient to allow for palm clearance beneath the pelvis; grade 2, elevation sufficient to allow for fist clearance for fewer than five repetitions; and grade 3, elevation sufficient to allow for fist clearance for five or more repetitions. Hand grip strength was measured using a Jamar hydraulic dynamometer, and knee extensor strength was assessed using a handheld dynamometer according to standardized positioning protocols [26]. Cognitive function and symptom burden were evaluated using the Korean version of the Mini-Mental State Examination (MMSE) [27], and pain severity was assessed using the Numeric Rating Scale (NRS).

Health-related quality of life was assessed using the EuroQol-5 Dimension (EQ-5D) instrument and the EuroQol Visual Analog Scale (EQ-VAS) [28]. The EQ-5D evaluates five domains of health status: mobility, self-care, usual activities, pain/discomfort, and anxiety/depression. The EQ-VAS records patients’ self-rated overall health on a vertical visual analog scale ranging from 0 (worst imaginable health state) to 100 (best imaginable health state).

### 2.4. Statistical Analysis

Continuous variables are presented as the mean ± standard deviation, and categorical variables as counts and percentages. Baseline demographic and clinical characteristics were compared between the high-compliance (≥50%) and low-compliance (<50%) groups using independent two-sample *t*-tests for continuous variables and chi-square or Fisher’s exact test for categorical variables, as appropriate. Longitudinal changes in functional outcomes, pain scores, and %HRR across three time points (baseline, Week 1, and Week 2) were analyzed using linear mixed-effects models with random intercepts to account for within-subject correlations. Fixed effects included time, compliance group, and the interaction between time and compliance group (time × compliance). The primary parameters of interest were the main effect of time, the main effect of compliance group, and the interaction term to determine whether temporal changes differed according to compliance level. When a significant effect of compliance or time × compliance interaction was identified, post hoc pairwise comparisons were conducted to examine (1) within-group changes across time points (baseline vs. Week 1, baseline vs. Week 2, and Week 1 vs. Week 2) and (2) between-group differences at each time point. Estimated marginal means and contrasts were calculated using the emmeans package with Bonferroni adjustment for multiple comparisons. Changes in exercise program progression variables, including exercise step level and applied resistance intensity, were analyzed using the Friedman test to evaluate within-subject differences across time points. Linear mixed-effects models were employed as they are robust for longitudinal data with small sample sizes, effectively accounting for within-subject correlations and individual variability through random intercepts. However, given the pilot nature of this study and the limited size of the high-compliance group (n = 7), the results of the interaction effects (time × compliance) were considered exploratory rather than definitive. Model-based estimated marginal means with 95% confidence intervals from the linear mixed-effects models were plotted to illustrate longitudinal trajectories according to compliance group. All statistical analyses were performed using R software (version 4.3.0; R Foundation for Statistical Computing, Vienna, Austria). A two-sided *p*-value of <0.05 was considered statistically significant.

## 3. Results

A total of 25 patients were initially enrolled in this pilot study. Five patients (20.0%) dropped out during the 2-week intervention period for the following reasons: one patient was readmitted to the ICU due to medical status changes, three patients were transferred to other hospitals during the intervention, and one patient refused to continue the program. Consequently, 20 patients successfully completed the final follow-up assessments and were included in the primary analysis, yielding a retention rate of 80.0%. Across the 20 analyzed patients, the overall mean rehabilitation compliance was 40.6% (34.8% at Week 1 and 46.4% at Week 2). Based on rehabilitation compliance, 13 patients (65.0%) were classified into the low-compliance group (<50%), and seven patients (35.0%) into the high-compliance group (≥50%). No serious adverse events related to the mobile application intervention were reported throughout the study period.

The mean age was 52.7 ± 13.9 years, and nine participants (45.0%) were male (Table 1). Liver transplantation was the most common primary diagnosis (n = 14, 70.0%), followed by lung transplantation (n = 6, 30.0%). A comparison of baseline characteristics by compliance status revealed that patients in the high-compliance group had significantly longer total hospital length of stay and ICU stay compared with those in the low-compliance group (Table 1). No significant between-group differences were observed in age, sex, body mass index, diagnosis category, comorbidities, duration of mechanical ventilation, or caregiver presence during hospitalization.

**Figure 2:**
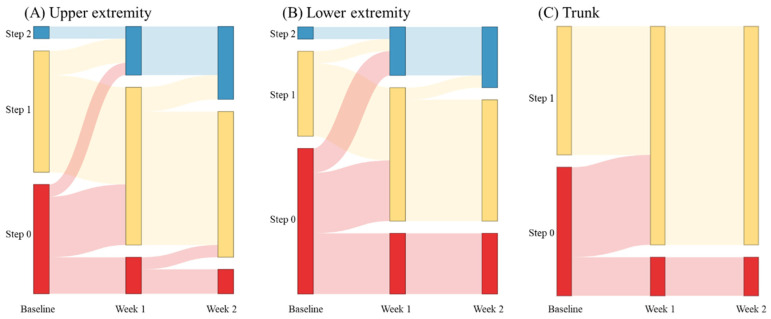
Exercise program progression over time for (**A**) upper extremity, (**B**) lower extremity, and (**C**) trunk components. Colors indicate exercise step levels: red = Step 0, yellow = Step 1, blue = Step 2.

Exercise program parameters demonstrated progressive increases in training intensity and exercise level over the intervention period (Table 2). Step levels for upper extremity, lower extremity, and trunk exercises significantly increased from baseline to Week 1 and Week 2. Applied resistance intensity similarly increased over time, reflecting gradual advancement in training difficulty. Figure 2 illustrates the longitudinal transition of exercise levels, demonstrating consistent progression toward higher training stages throughout the intervention.

**Figure 3:**
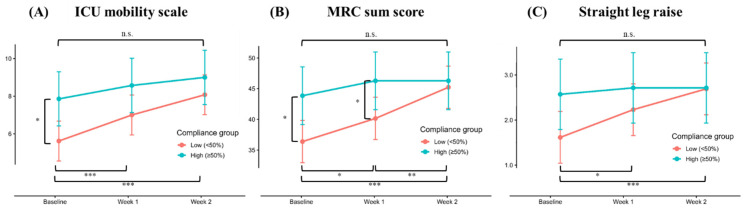
Longitudinal trajectories of functional outcomes by compliance group. Estimated marginal means with 95% confidence intervals for (**A**) ICU Mobility Scale, (**B**) MRC-SS, and (**C**) straight leg raise across baseline, Week 1, and Week 2, stratified by rehabilitation compliance (low < 50% vs. high ≥ 50%). Brackets indicate post hoc pairwise comparisons within each compliance group across time points, as well as between compliance groups at each time point. Statistical significance for post hoc pairwise comparisons is denoted as *: *p* < 0.05, **: *p* < 0.01, and ***: *p* < 0.001. No significant differences were observed between time points in the high-compliance group (denoted as n.s.).

Across the overall cohort, most functional outcome measures improved significantly over time. The MRC-SS, ICU Mobility Scale, FAC, grip strength, bridge, SLR, MMSE, EQ-5D, EQ-VAS, and NRS scores showed significant main effects of time (all *p* < 0.05) (Table 3). The mean improvements in the MRC-SS (6.6 points) and ICU Mobility Scale (2.0 points) met the established minimal clinically important difference. Marginal between-group differences were observed for MRC-SS and SLR, whereas the ICU Mobility Scale and bridge demonstrated a significant main effect of compliance (Table 3). A significant time × compliance interaction effect was identified for MRC-SS and SLR, indicating differential recovery trajectories between compliance groups. %HRR did not demonstrate a statistically significant change over time, although a modest increasing trend was observed. The recorded %HRR values corresponded to very light to light exercise intensity [29]. Figure 3 presents the model-based estimated marginal mean trajectories for the ICU Mobility Scale, MRC-SS, and SLR stratified by compliance group. Post hoc analyses showed that, in the low-compliance group, the ICU Mobility Scale improved significantly from baseline to Week 1 and from baseline to Week 2. Significant within-group improvements across all pairwise time comparisons were also observed for MRC-SS and SLR in the low-compliance group. In contrast, no statistically significant within-group changes were detected in the high-compliance group for these outcomes. Between-group comparisons revealed a significant difference in the ICU Mobility Scale at baseline, but not at Weeks 1 or 2. For MRC-SS, significant between-group differences were observed at baseline and Week 1, but not at Week 2. No significant between-group differences were observed for SLR at any time point.

Overall patient satisfaction and perceived effectiveness of the mobile application-based rehabilitation program were high (Table 4). Most questionnaire items yielded mean scores exceeding 4.3 on the 5-point Likert scale, indicating favorable acceptability, perceived benefit, usability, and safety. Perceived exercise intensity was rated at approximately 3 points, suggesting that participants considered the training load appropriate and manageable.

## 4. Discussion

This study demonstrates that a mobile application-based rehabilitation program is feasible and associated with clinically meaningful functional improvements in non-ambulatory patients during the early ward phase following ICU discharge. However, since this study lacks a control group, these improvements may reflect a combination of the intervention, spontaneous recovery, and the cumulative effects of standard patient care. Improvements were observed across multiple domains, including muscle strength, mobility, ambulation category, cognition, and health-related quality of life. Notably, the significant gains observed in the low-compliance group likely reflect their more severe initial functional impairment, where even partial participation leads to measurable change. In contrast, the high-compliance group’s recovery was likely tempered by higher clinical complexity, evidenced by their longer ICU stays. However, the observed increase in compliance from Week 1 (34.8%) to Week 2 (46.4%) suggests that as patients’ physical capacity improved and familiarity with the digital interface increased, engagement with the intervention became more sustainable. The absence of significant increases in %HRR despite functional improvement indicates that the program was physiologically tolerable, remaining within very light to light intensity ranges. High patient satisfaction and favorable usability ratings further support the acceptability and safety of this approach. Collectively, these findings suggest that individualized, mobile application-based rehabilitation may represent a scalable and pragmatic strategy to initiate early mobilization and functional recovery in deconditioned post-ICU patients.

Extensive evidence from ICU rehabilitation trials and systematic reviews supports the role of early mobilization and structured exercise in reducing ICUAW and improving downstream functional outcomes with an acceptable safety profile [6,7]. Systematic reviews and meta-analyses have consistently reported reductions in ICUAW incidence and improvements in functional mobility [30]. However, despite this robust evidence base, implementation in routine clinical practice remains inconsistent. Notable barriers—including limited staffing, a lack of specialized equipment, and organizational constraints—continue to impede the delivery of timely and structured rehabilitation [31,32]. Resource limitations have been identified as primary obstacles to providing consistent, high-quality rehabilitation in both ICU and post-ICU settings.

Against this backdrop, our findings suggest that a structured, progressive, and personalized exercise regimen delivered via a smartphone application may offer a pragmatic strategy to address these implementation barriers. By providing a scalable platform that requires clinician oversight but no specialized heavy equipment, the MORA system was successfully implemented in deconditioned patients. This approach was associated with meaningful gains in strength, mobility, ambulation category, cognition, and health-related quality of life, even within a resource-constrained environment. Our observation of improved mobility and functional categories aligns with a recent prospective pilot trial on general surgical wards, which demonstrated that extending post-ICU physiotherapy resources significantly enhanced specific mobility domains, such as bed-to-chair transfers and stepping performance [33]. However, while expanding physical therapy staffing or frequency can successfully drive functional recovery, its widespread implementation is frequently hindered by institutional resource limitations. Although our mobile application-guided approach circumvents these staffing constraints by offering a highly scalable alternative, the low exercise intensity achieved in this study necessitates caution.

Given that the exercise intensity measured by %HRR remained in the very light to light range, the training load was likely insufficient to induce true muscle hypertrophy. Therefore, the observed gains in MRC-SS and grip strength likely reflect neural adaptations, neuromuscular familiarization, or spontaneous post-ICU recovery rather than physiological muscle adaptations directly induced by the application. This line of reasoning is consistent with a recent large-scale randomized controlled trial evaluating neuromuscular electrical stimulation combined with personalized physiotherapy post-ICU discharge; that study similarly found no significant differences in global muscle strength at hospital discharge across the overall cohort, though it suggested potential localized benefits specifically for patients with established ICU-acquired weakness [34]. In addition, while the initial five days of supervised physical therapy may have introduced a Hawthorne effect or provided essential guidance [35], the observed increase in compliance from Week 1 to Week 2 suggests a transition toward user adaptation and the independent utility of the application. Moreover, our hybrid approach aligns with recent evidence suggesting that this may offer higher engagement and feasibility than traditional in-person follow-ups [11], effectively bridging the gap between clinical oversight and sustainable rehabilitation.

The predominance of low compliance in this study is clinically plausible and should be interpreted within the broader context of post-ICU survivorship. While traditional in-person physical therapy faces substantial logistical and workforce barriers, mobile application-based rehabilitation offers a distinct advantage by providing a scalable, low-resource solution that ensures therapeutic continuity without the constant physical presence of a therapist [14]. Nevertheless, even with this approach, patient-level barriers persist, including fluctuating cognition, limited digital literacy, physical fatigue, and the need for caregiver assistance [36,37]. Despite these challenges, the numerical increase in compliance observed during the study period is encouraging. This trend suggests that the inpatient setting may provide a strategic advantage for mobile application-based rehabilitation. Unlike outpatients, who must independently navigate digital platforms, hospitalized patients benefit from initial clinician-led instruction and immediate support from nurses or caregivers. Accordingly, the application of this rehabilitation in hospitalized patients with ICUAW is particularly meaningful: the structured hospital environment may mitigate inherent barriers to digital engagement while maximizing recovery potential among highly vulnerable survivors.

Static educational materials alone are insufficient to ensure adherence, as they lack the interactive and iterative components required for complex physical recovery [38,39]. Similarly, uniform, non-tailored exercise videos are often inappropriate for post-ICU populations, given the marked interpatient variability in physical and cognitive status [40]. In contrast, our study underscores the feasibility of a personalized mobile application-based rehabilitation model. Rather than providing a generic set of exercises, specialized physiatrists prescribed individualized programs through the MORA system, carefully tailored to each patient’s functional baseline and bed-level capacity. Although the application itself does not possess real-time kinematic correction capabilities, exercise quality and safety were ensured through the following process. Initial in-person sessions with a physical therapist established correct movement patterns, while the platform’s ability to increment intensity via systematic posture changes and external resistance (sandbags) allowed for precise, clinician-monitored dose management.

Although this single-arm study cannot establish causal effectiveness or superiority over traditional rehabilitation methods, the successful implementation of such a tailored, application-guided approach in severely deconditioned patients demonstrates its clinical feasibility and safety. This study provides a foundational framework for future randomized controlled trials, which are necessary to control for confounding factors such as regression to the mean and natural post-ICU recovery. Looking forward, future iterations of mobile application-based rehabilitation could integrate bedside infrastructure and wearable sensors. By replacing manual monitoring with real-time acquisition of objective physiological data, clinicians can further refine personalized exercise prescriptions. Such technological integration may enhance scalability and efficiency while supporting the safe delivery of higher-intensity, individualized rehabilitation, thereby transforming the general ward into a proactive recovery environment.

## 5. Limitations

This study has several limitations that warrant consideration. First, the single-arm design without a control group limits causal inference regarding the effectiveness of the intervention. The functional gains observed may be influenced by spontaneous recovery, natural post-ICU stabilization, or standard nursing care. Without a direct comparison, the specific therapeutic contribution of the mobile application remains preliminary. Second, the relatively small sample size and single-center setting may reduce statistical power and limit the generalizability to other clinical contexts. Third, the absence of long-term follow-up precludes evaluation of the durability of functional improvements and the sustainability of exercise adherence after hospital discharge. Fourth, the clinical assessments were not performed by a blinded independent rater, which may have introduced observer bias during the evaluation of functional outcomes. Although standardized assessment protocols were strictly followed to ensure consistency, the open-label nature of this pilot study represents a limitation in the interpretation of functional recovery. Furthermore, the specific grading scales used for evaluating SLR and bridge performance were custom-designed internally for the pragmatic purposes of this pilot trial and lack formal, external validation in the ICUAW population.

## 6. Conclusions

This study demonstrates that a personalized, mobile application-based rehabilitation program is feasible and safe to implement in severely deconditioned and non-ambulatory patients during the early ward phase following ICU discharge. While participants showed significant improvements across multiple domains, including muscle strength, mobility, ambulation capacity, cognitive function, and health-related quality of life, these observed changes cannot be definitively attributed to the intervention itself due to the lack of a control group. Through tailored interventions from a specialized exercise library, progressive advancement of training was successfully achieved, accompanied by high patient satisfaction. However, the potential for spontaneous recovery and the influence of standard inpatient care must be considered when interpreting these preliminary functional outcomes. Despite these limitations, the high acceptability and safety of this digital approach suggest that it may serve as a practical adjunct to bridge rehabilitation gaps. Rigorous, randomized controlled trials are essential to differentiate the independent effects of this mobile application-based rehabilitation intervention from natural post-ICU recovery and to confirm its clinical effectiveness.

## Figures and Tables

**Table 1:** Demographics of study population (n = 20).

Variables	Mean ± SD or n (%)	Compliance < 50% (n = 13)	Compliance ≥ 50% (n = 7)	*p*-Value
Age	52.7 ± 13.9	51.2 ± 14.0	55.4 ± 14.5	0.534
Sex				0.742
Male	9 (45.0%)	5 (38.5%)	4 (57.1%)	
Female	11 (55.0%)	8 (61.5%)	3 (42.9%)	
Body mass index	19.8 ± 3.6	20.0 ± 3.3	19.4 ± 4.4	0.768
Diagnosis				0.152
Liver transplantation	14 (70.0%)	11 (84.6%)	3 (42.9%)	
Lung transplantation	6 (30.0%)	2 (15.4%)	4 (57.1%)	
Medical history				
Hypertension	2 (10.0%)	2 (15.4%)	0 (0.0%)	0.755
Diabetes mellitus	8 (40.0%)	5 (38.5%)	3 (42.9%)	1.000
Cardiac disease	1 (5.0%)	0 (0.0%)	1 (14.3%)	0.747
Chronic kidney disease	0 (0.0%)	0 (0.0%)	0 (0.0%)	n/a
Cerebrovascular disease	2 (10.0%)	1 (7.7%)	1 (14.3%)	1.000
Cancer	1 (5.0%)	0 (0.0%)	1 (14.3%)	0.747
Hospital stay, days	95.7 ± 43.2	81.4 ± 37.7	122.1 ± 42.5	0.040
ICU stay, days	21.2 ± 12.3	16.6 ± 9.9	29.6 ± 12.6	0.020
Mechanical ventilation days	19.7 ± 10.8	16.7 ± 9.9	25.1 ± 10.8	0.095
Caregiver				1.000
Family	15 (75.0%)	10 (76.9%)	5 (71.4%)	
Non-family	5 (25.0%)	3 (23.1%)	2 (28.6%)	

ICU: intensive care unit.

**Table 2:** Exercise program and intensity.

		Baseline	Week 1	Week 2	*p*-Value
Upper extremity					
Step	0	9 (45.0%)	3 (15.0%)	2 (10.0%)	<0.001
	1	10 (50.0%)	13 (65.0%)	12 (60.0%)	
	2	1 (5.0%)	4 (20.0%)	6 (30.0%)	
Weight, kg	0	9 (45.0%)	5 (25.0%)	4 (20.0%)	<0.001
	0.5	9 (45.0%)	10 (50.0%)	9 (45.0%)	
	1–1.5	2 (10.0%)	5 (25.0%)	7 (35.0%)	
Lower extremity					
Step	0	12 (60.0%)	5 (25.0%)	5 (25.0%)	<0.001
	1	7 (35.0%)	11 (55.0%)	10 (50.0%)	
	2	1 (5.0%)	4 (20.0%)	5 (25.0%)	
Weight, kg	0	15 (75.0%)	12 (60.0%)	12 (60.0%)	0.076
	0.5–1	4 (20.0%)	5 (25.0%)	6 (30.0%)	
	1.5–2	1 (5.0%)	3 (15.0%)	2 (10.0%)	
Trunk					
Step	0	10 (50.0%)	3 (15.0%)	3 (15.0%)	<0.001
	1	10 (50.0%)	17 (85.0%)	17 (85.0%)	

**Table 3:** Changes in functional evaluation, pain, and heart rate reserve.

	Baseline	Week 1	Week 2	Adjusted *p* for Time	Adjusted *p* for Compliance	Adjusted *p* for Time × Compliance
MRC-SS	39.0 ± 7.7	42.3 ± 6.1	45.6 ± 5.5	<0.001	0.089	0.011
ICU mobility scale	6.4 ± 2.5	7.5 ± 2.0	8.4 ± 1.3	<0.001	0.019	0.181
FAC	1.1 ± 1.0	1.9 ± 1.2	2.2 ± 1.1	<0.001	0.613	0.161
Grip						
Right	9.8 ± 7.1	10.9 ± 6.4	12.4 ± 6.3	0.001	0.370	0.505
Left	8.8 ± 7.1	11.0 ± 6.0	11.6 ± 6.8	0.004	0.845	0.107
Bridge	1.6 ± 1.3	2.0 ± 1.1	2.4 ± 1.0	<0.001	0.044	0.069
SLR	1.9 ± 1.2	2.4 ± 1.1	2.7 ± 0.7	0.002	0.061	0.024
MMSE	27.6 ± 1.7	27.9 ± 1.5	28.6 ± 1.6	0.005	0.561	0.248
EQ-5D	0.44 ± 0.22	0.44 ± 0.24	0.57 ± 0.23	0.005	0.543	0.444
EQ-VAS	52.7 ± 21.8	66.8 ± 18.2	68.7 ± 18.6	0.002	0.324	0.448
NRS	1.6 ± 2.0	0.6 ± 1.3	0.8 ± 1.2	0.005	0.697	0.734
%HRR	23.3 ± 13.6	23.5 ± 13.7	26.0 ± 16.3	0.681	0.717	0.673

Adjusted for age, sex, diagnosis, and ICU length of stay. EQ-5D: EuroQol-5 Dimension; EQ-VAS: the EuroQol Visual Analog Scale; FAC: Functional Ambulatory Category; HRR: Heart rate reserve; ICU: Intensive care unit; MMSE: Mini-Mental State Examination; MRC-SS: Medical Research Council Sum Score; NRS: Numeric Rating Scale; SLR: Straight leg raise.

**Table 4:** Questionnaires.

	Week 1	Week 2
** Satisfaction **		
During the past five days, on how many days did you actually use the application?	4.2 ± 1.1	3.7 ± 1.6
How satisfied are you overall with the application?	4.4 ± 0.6	4.3 ± 0.9
Did your interest in your own health increase after participating in the exercise program?	4.4 ± 0.8	4.2 ± 1.0
Would you be willing to continue using this program in the future?	4.3 ± 0.7	4.2 ± 1.0
** Perceived Effectiveness **		
Overall, do you think the exercise program was effective?	4.4 ± 0.6	4.4 ± 0.8
Do you think your muscle strength improved after participating in the exercise program?	4.2 ± 0.9	4.4 ± 0.9
Do you think your walking ability improved after participating in the exercise program?	4.3 ± 1.0	4.1 ± 1.0
Did your confidence in your recovery improve after participating in the exercise program?	4.3 ± 0.9	4.1 ± 0.9
** Application-Related Experience **		
Was the application service easy to use?	4.3 ± 0.9	4.6 ± 0.6
Did the application function smoothly without technical problems?	4.3 ± 0.8	4.5 ± 0.7
Did your trust in the hospital providing the exercise program increase after using the application?	4.4 ± 1.0	4.3 ± 0.7
** Safety **		
Was the exercise program safe overall?	4.7 ± 0.6	4.6 ± 0.7
** Intensity **		
How would you rate the intensity of the exercise program provided?	3.0 ± 0.8	3.1 ± 0.5

All items were rated on a 5-point Likert scale. For satisfaction, effectiveness, usability, and safety items: 1 = strongly disagree, 2 = disagree, 3 = neutral, 4 = agree, and 5 = strongly agree. For exercise intensity: 1 = very easy, 2 = easy, 3 = appropriate, 4 = difficult, and 5 = very difficult.

## Data Availability

The datasets analyzed during the current study are not publicly available due to restrictions under local data protection regulations and institutional review board (IRB) policies regarding patient confidentiality. However, the data are available from the corresponding author upon reasonable request and subject to institutional approval.

## Supplementary Materials

The following supporting information can be downloaded at: https://www.mdpi.com/article/10.3390/jcm15114211/s1.
