# Supplementary material for: The Feasibility, Safety, and Preliminary Functional Outcomes of a Mobile Application-Based Rehabilitation Program in Non-Ambulatory Patients After Intensive Care Unit Discharge
Source: J Clin Med. 2026 May 29;15(11):4211. doi: 10.3390/jcm15114211 (PMC13257730; doi:10.3390/jcm15114211)

Supplementary materials

1. Stepwise exercise program

A) Upper extremity exercise program

Step 0

1) Elbow flexion

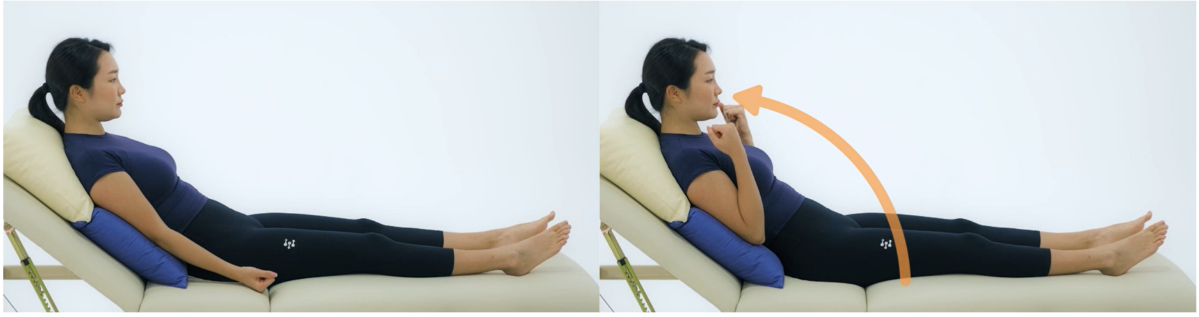

2) Forward arm raise

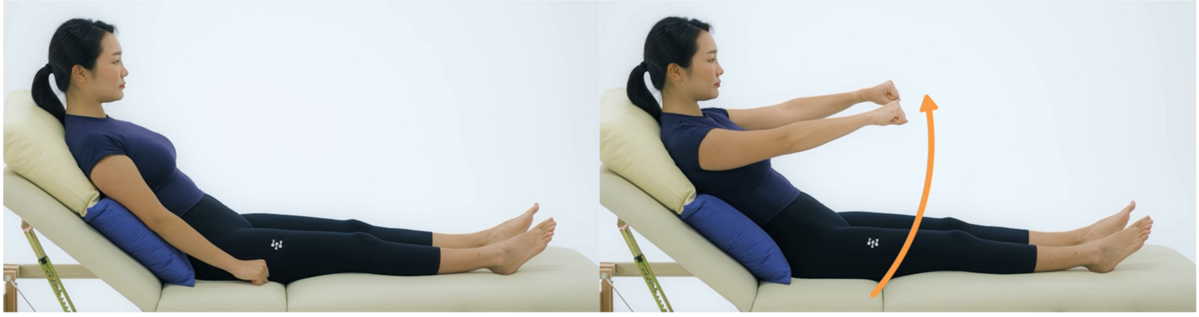

3) Chest press

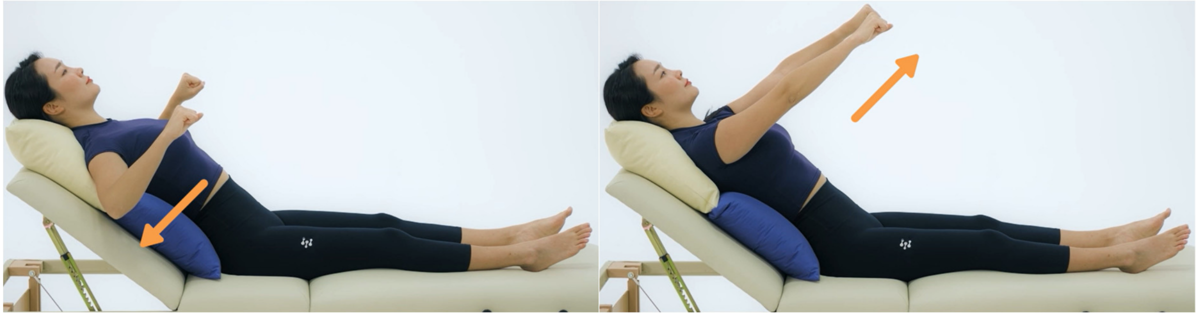

Step 1

1) Chest fly with elbow extension

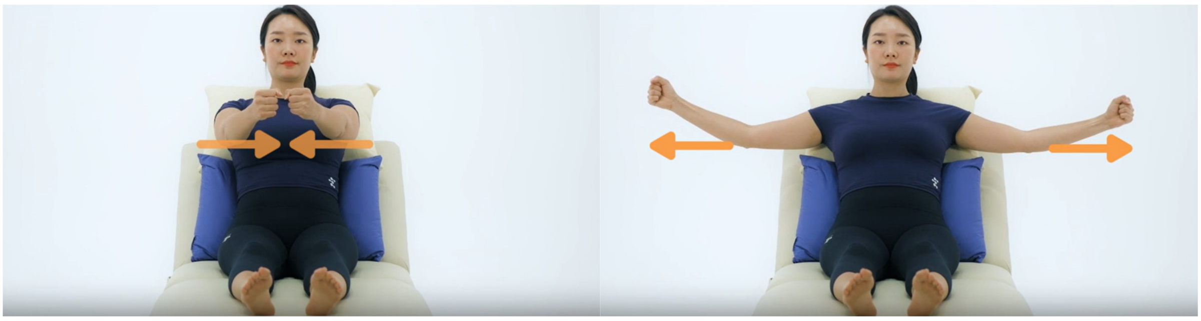

2) Shoulder press

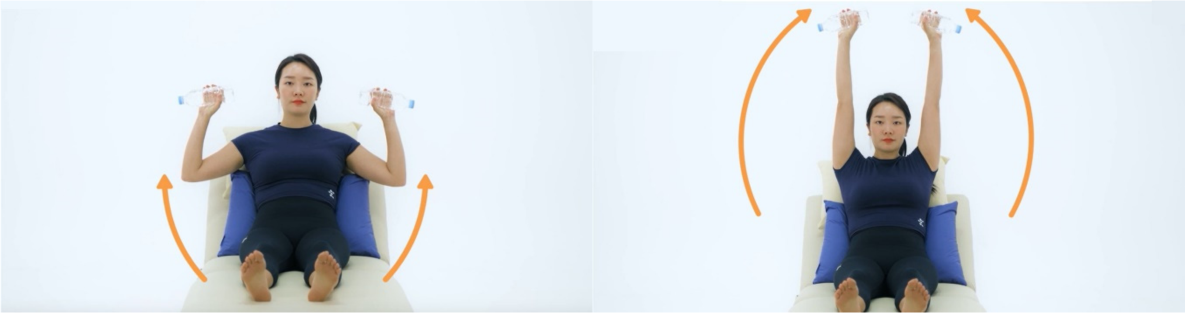

3) Shoulder forward flexion

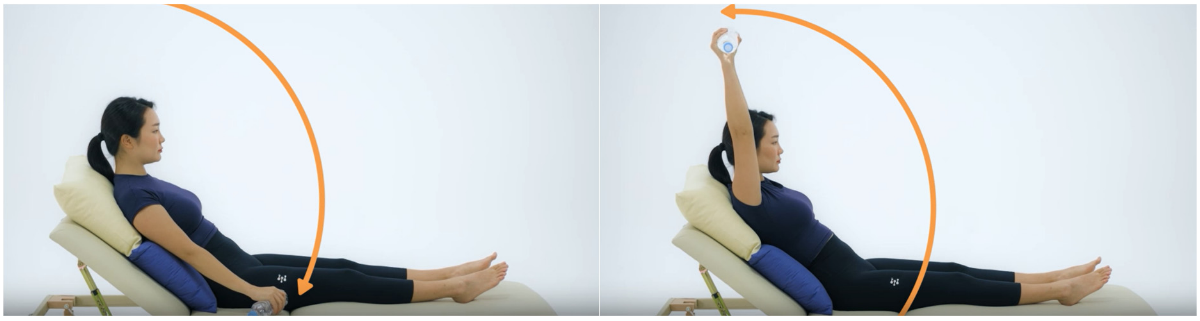

4) Rowing

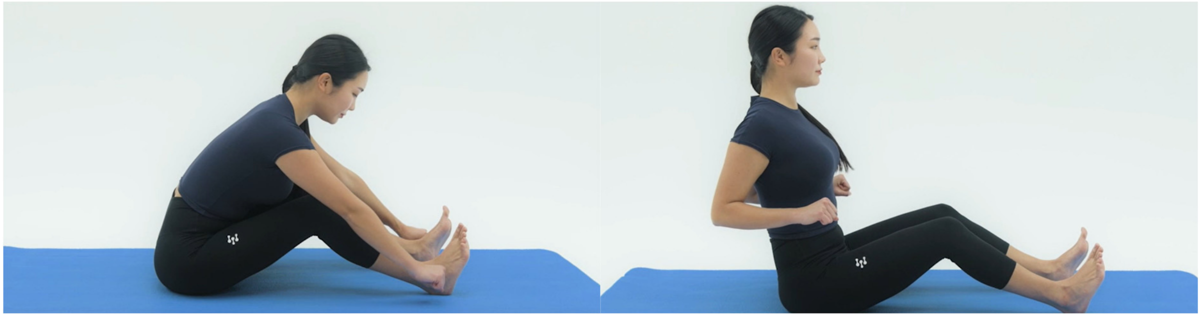

Step 2

1) Seated elbow flexion

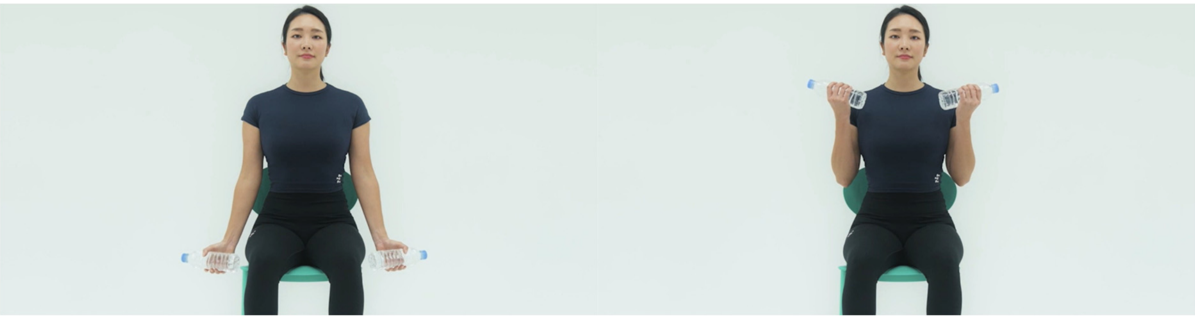

2) Shoulder shrugs

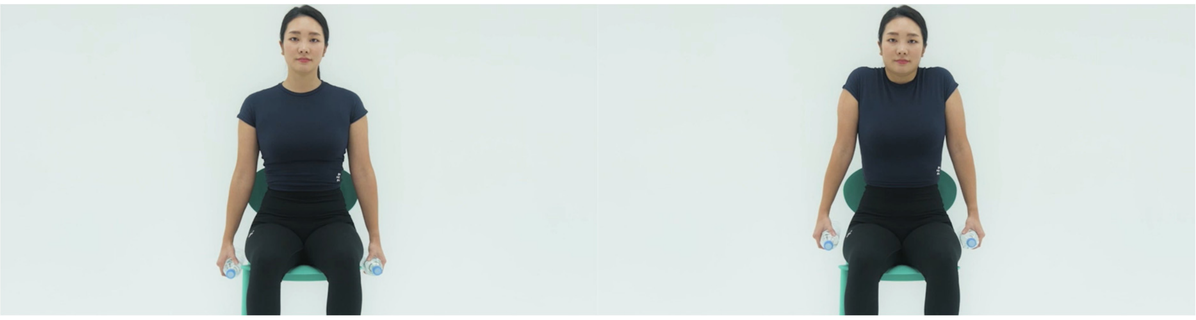

3) Arm lifting

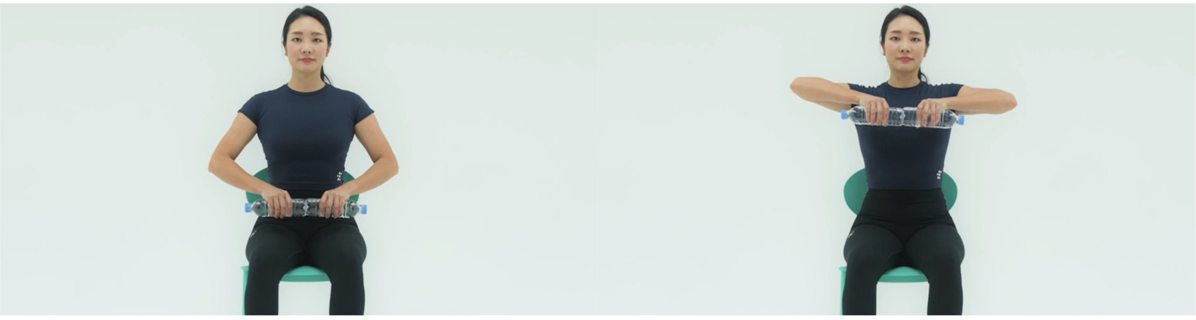

4) Overhead arm reach

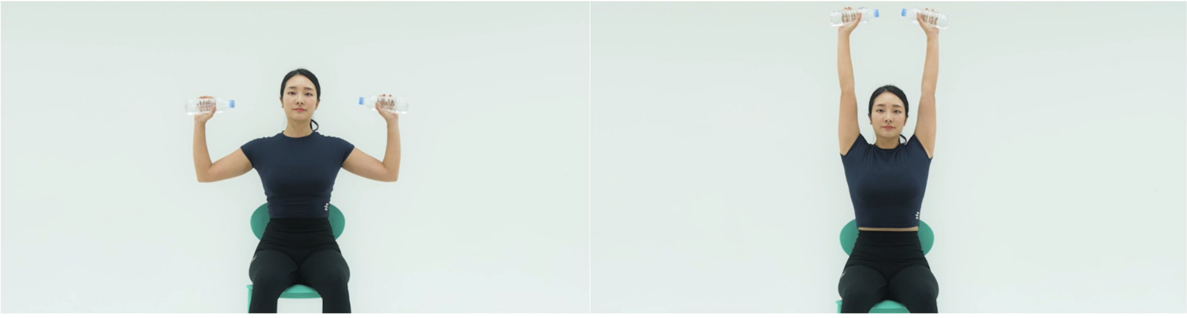

5) Shoulder abduction

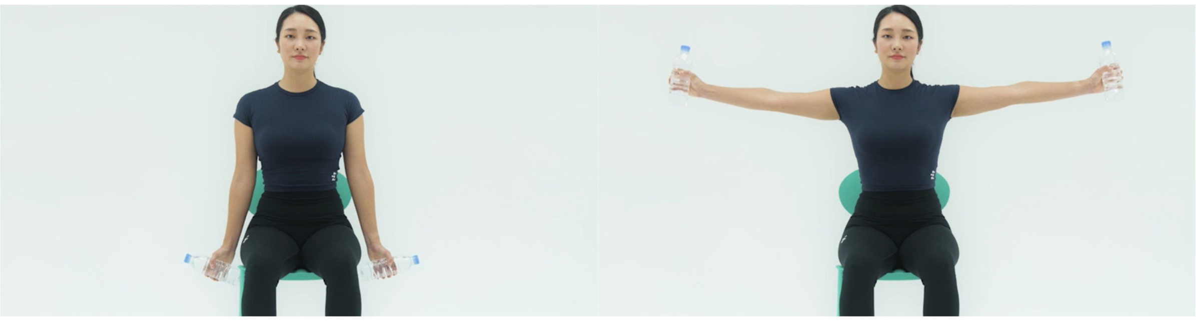

B) Lower extremity exercise program

Step 0

1) Alternating ankle pump

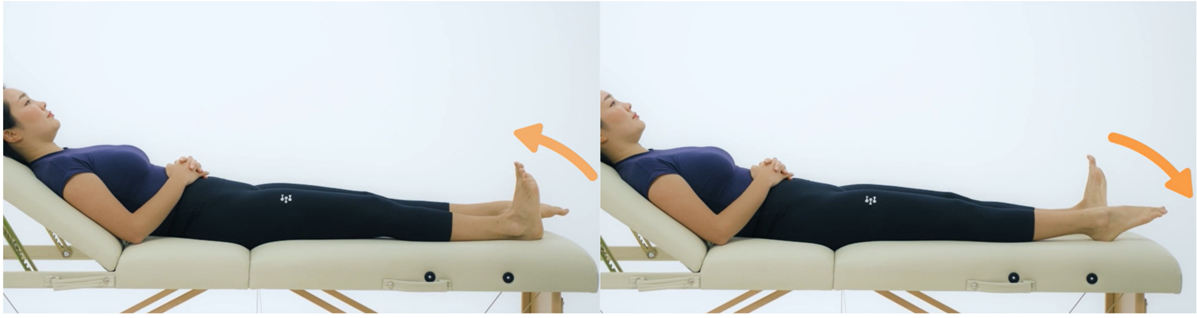

2) Knee extension

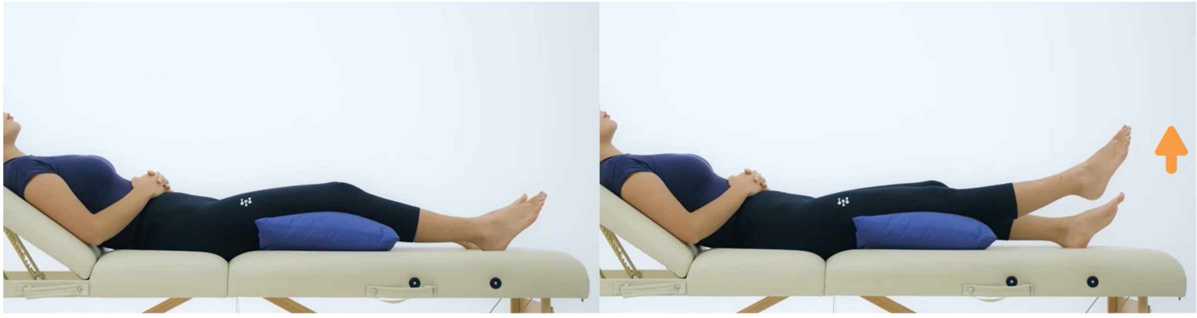

3) Clamshell

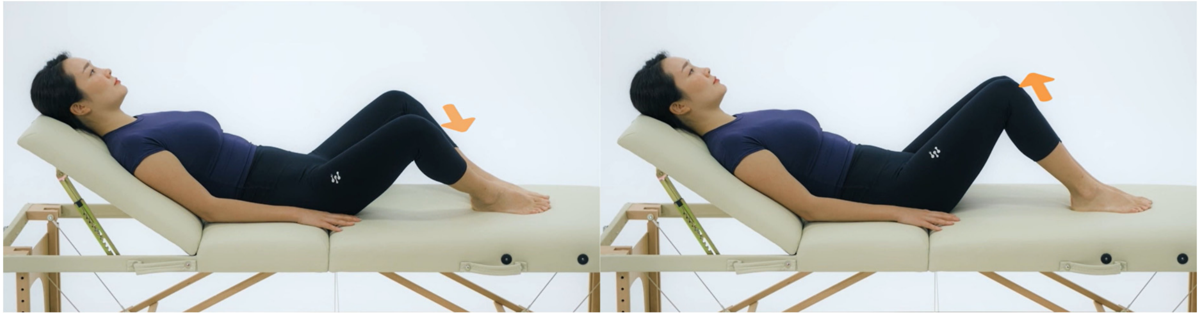

Step 1

1) Hip and knee flexion

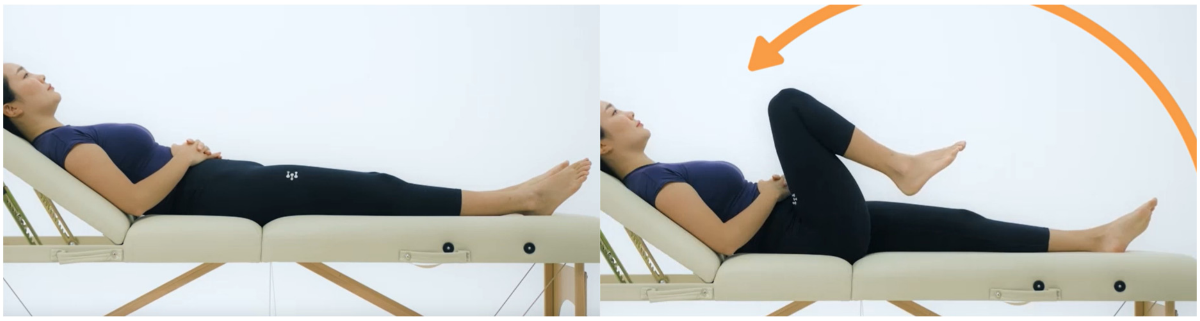

2) Straight leg raise with knee extension

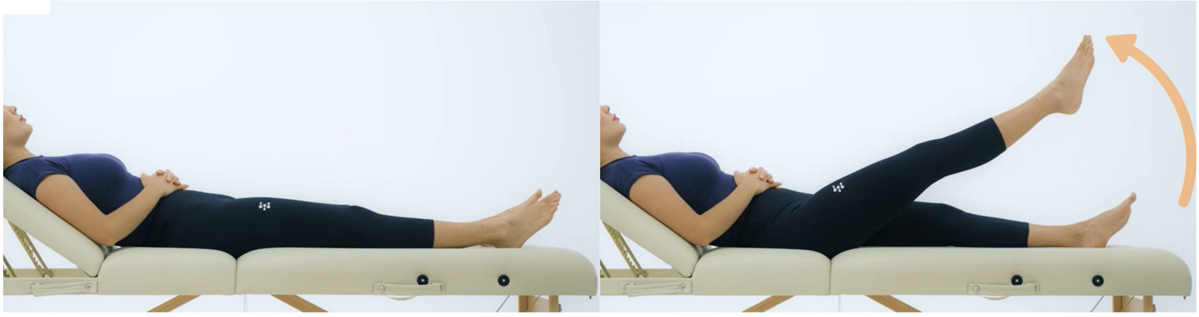

Step 2

1) Seated ankle pump

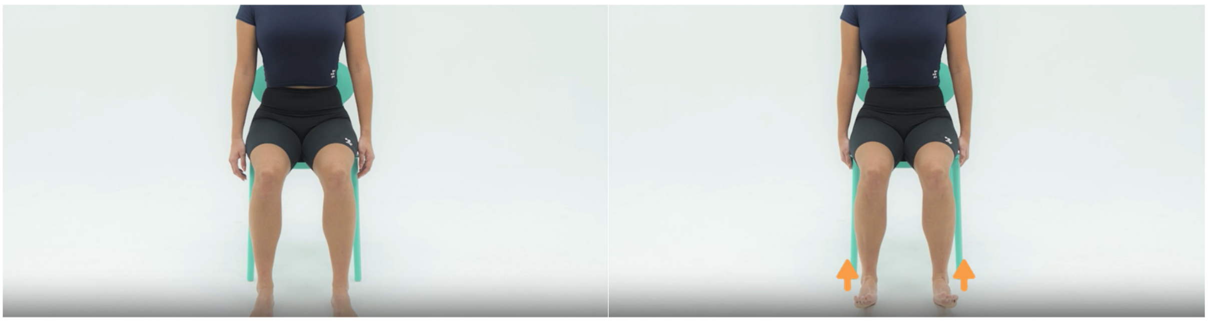

2) Seated knee extension

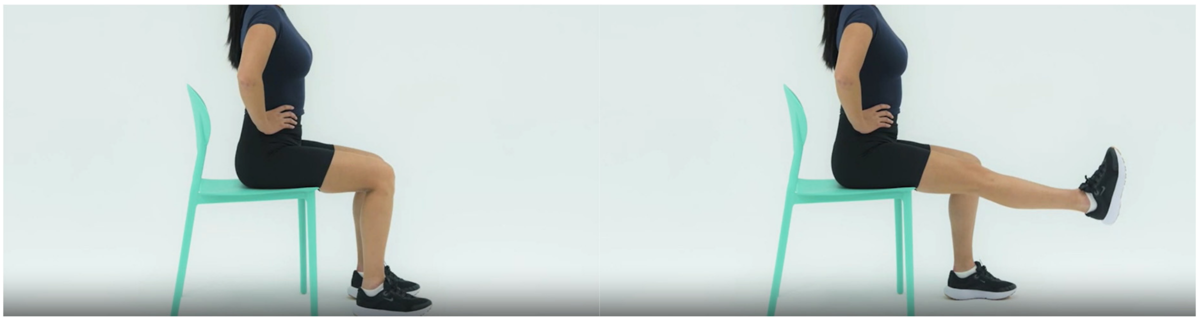

3) Seated hip flexion

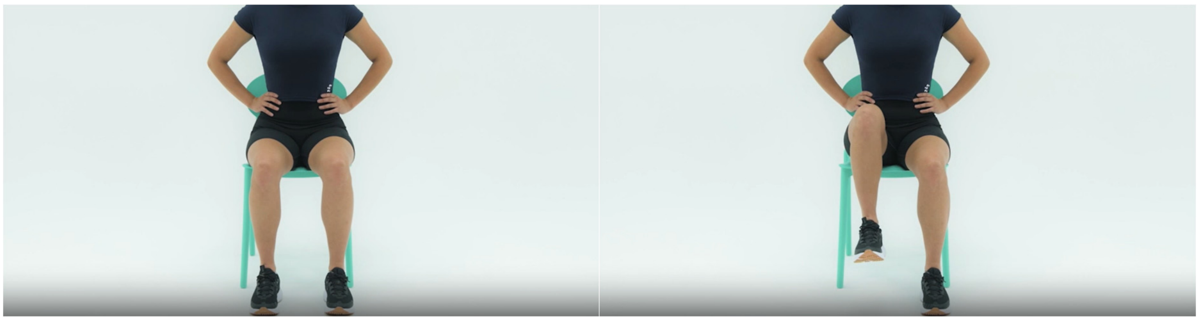

4) Seated leg raise hold

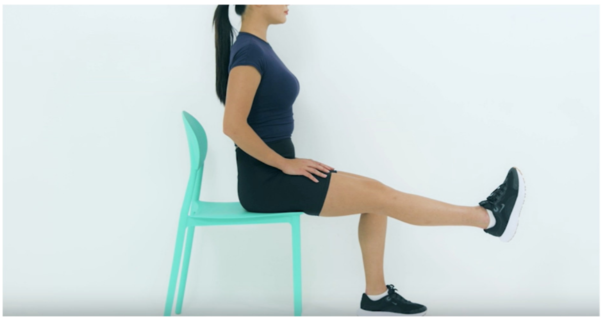

5) Scissor kicks

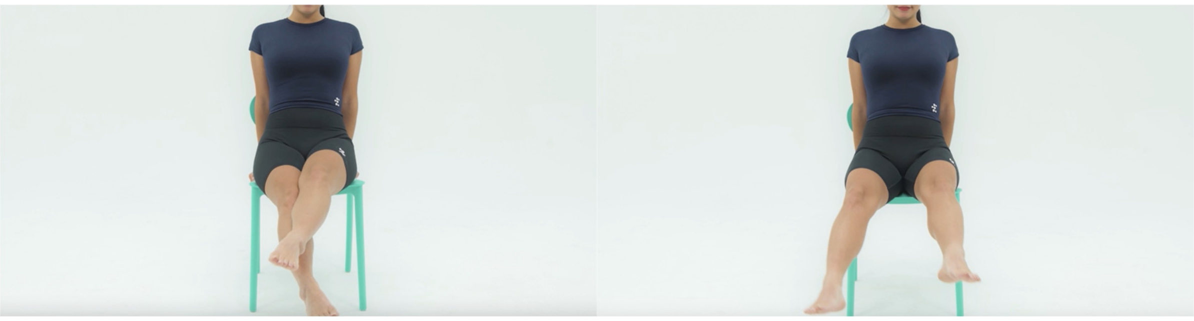

C) Trunk exercise program

Step 0

1) Supine pelvic bridge

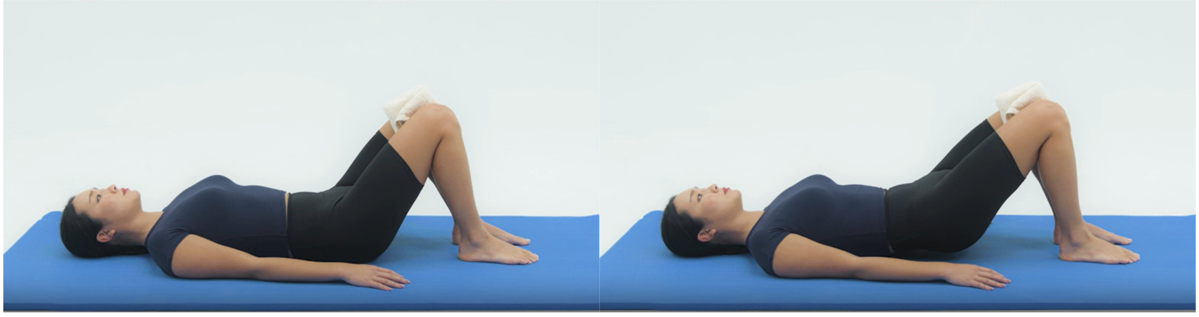

Step 1

1) Partial sit-up hold

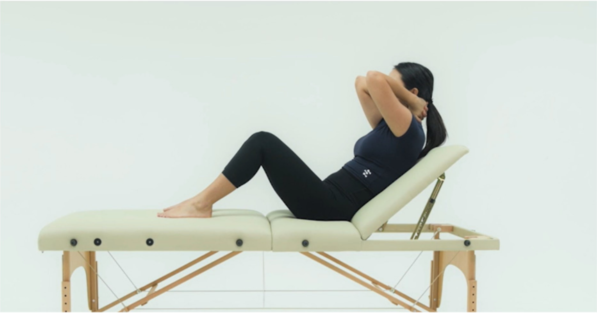

2) Full hip bridge

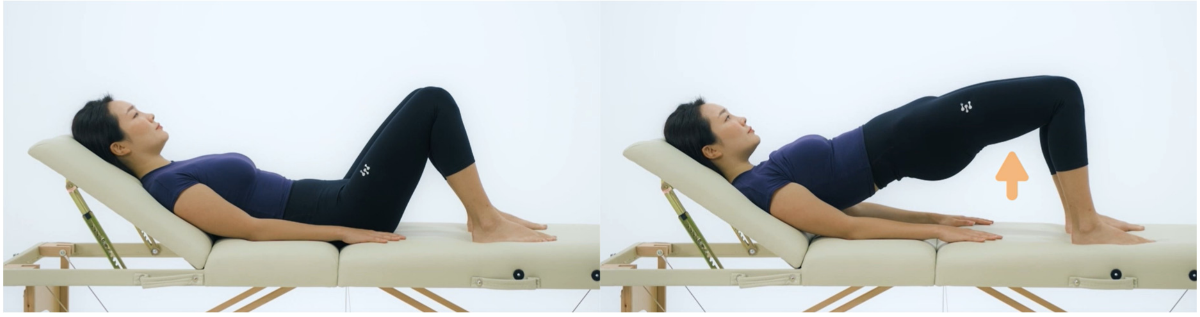

Supplement: Supplementary file 1 [file jcm-15-04211-s001.zip › jcm-4327312-supplementary.pdf]
